# Development of a Double Nuclear Gene-Targeting Method by Two-Step Transformation Based on a Newly Established Chloramphenicol-Selection System in the Red Alga *Cyanidioschyzon merolae*

**DOI:** 10.3389/fpls.2017.00343

**Published:** 2017-03-14

**Authors:** Takayuki Fujiwara, Mio Ohnuma, Tsuneyoshi Kuroiwa, Ryudo Ohbayashi, Shunsuke Hirooka, Shin-Ya Miyagishima

**Affiliations:** ^1^Department of Cell Genetics, National Institute of GeneticsShizuoka, Japan; ^2^Japan Science and Technology Agency, Core Research for Evolutional Science and TechnologySaitama, Japan; ^3^Department of Genetics, Graduate University for Advanced StudiesShizuoka, Japan; ^4^National Institute of Technology, Hiroshima CollegeHiroshima, Japan; ^5^Department of Chemical and Biological Science, Faculty of Science, Japan Women’s UniversityTokyo, Japan

**Keywords:** algae, plants, photosynthetic eukaryotes, genetic modification, multiple transformation, chloramphenicol, *CAT* selection marker

## Abstract

The unicellular red alga *Cyanidioschyzon merolae* possesses a simple cellular architecture that consists of one mitochondrion, one chloroplast, one peroxisome, one Golgi apparatus, and several lysosomes. The nuclear genome content is also simple, with very little genetic redundancy (16.5 Mbp, 4,775 genes). In addition, molecular genetic tools such as gene targeting and inducible gene expression systems have been recently developed. These cytological features and genetic tractability have facilitated various omics analyses. However, only a single transformation selection marker *URA* has been made available and thus the application of genetic modification has been limited. Here, we report the development of a nuclear targeting method by using chloramphenicol and the chloramphenicol acetyltransferase (*CAT*) gene. In addition, we found that at least 200-bp homologous arms are required and 500-bp arms are sufficient for a targeted single-copy insertion of the *CAT* selection marker into the nuclear genome. By means of a combination of the *URA* and *CAT* transformation systems, we succeeded in producing a *C. merolae* strain that expresses HA-cyclin 1 and FLAG-CDKA from the chromosomal *CYC1* and *CDKA* loci, respectively. These methods of multiple nuclear targeting will facilitate genetic manipulation of *C. merolae*.

## Introduction

Eukaryotic algae synthesize organic compounds such as carbohydrates and amino acids from inorganic materials by using light energy, and introduce organic products into aquatic ecosystems. Because, recent studies have revealed that microalgae (photosynthetic picoplankton) are the most abundant photosynthetic organisms in ocean ecosystems, studies of microalgae have become the major target of studies because these algae have major impact on ocean ecosystems (Worden et al., 2004). In addition, unicellular algae are potentially useful for other basic sciences in several different disciplines and applied fields for the following reasons: (1) cultures of unicellular algae often provide a homogeneous population of a single cell type, in contrast to land plants in which complicated cell and tissue differentiation systems have evolved, (2) in algal culture, each cell is exposed to a relatively homogeneous environment (light, inorganic nutrient concentrations, etc.), while each land plant cell is exposed to different environments, in the same individual, (3) unicellular eukaryotic algae often exhibit relatively simple cellular and genomic architectures, (4) the generation time required for unicellular algae is much shorter than for land plants, (5) unicellular algae are widespread in tree of life owing to secondary endosymbiotic events in which previously non-photosynthetic eukaryotes acquired chloroplasts through endosymbiotic association with eukaryotic algal endosymbionts (Reyes-Prieto et al., 2007), (6) unicellular algae are now being developed that are able to produce nutraceuticals and alternative energy (Radakovits et al., 2010). These characteristics of unicellular algae potentially offer ideal experimental platforms, but unfortunately, experimental tools, especially those for genetic modification of eukaryotic algae, have remained extremely limited.

The unicellular green alga *Chlamydomonas reinhardtii* has been widely used as a model eukaryotic alga because this alga is genetically tractable. However, there are still several difficulties in the genetic modification of this alga. The genome is very high in GC content (∼65%), which results in difficulties in PCR, reverse transcription and sequencing. Transgenes are integrated into the chromosomes randomly. Stable expression of heterologous proteins is difficult because of the high level of the activity of silencing transgenes (Rosales-Mendoza et al., 2012). In contrast to *C. reinhardtii*, procedures for gene targeting by homologous recombination have been developed in certain other eukaryotic algae, such as the green alga *Ostreococcus tauri* (Lozano et al., 2014) and the stramenopile *Nannochloropsis* sp. (Kilian et al., 2011). But instability of transgene expression because of gene silencing is still a serious problem (Tracey and Sohail, 2016).

In contrast to other eukaryotic algae that have yet to become genetically tractable, the unicellular red alga *Cyanidioschyzon merolae* has been developed as a promising model eukaryotic alga. Procedures for gene targeting by homologous recombination have been established. Transgenes are stably expressed without any silencing (Minoda et al., 2004; Fujiwara et al., 2013a). In addition, procedures for transient expression of a transgene (Ohnuma et al., 2008), an inducible transgene-expression system by heat-shock and inducible/repressible transgene expression systems by exchanging the nitrogen source in the medium have all been developed (Sumiya et al., 2014; Fujiwara et al., 2015). In addition to genetic tractability, *C. merolae* exhibits several other features that make it ideal for studies, as described below. The cellular content is very simple: the cell possesses a nucleus, one mitochondrion, one chloroplast, one peroxisome, one Golgi apparatus, one layer of ER and a minimal set of other single-membrane-bound organelles (Kuroiwa et al., 1998; Misumi et al., 2005). These organelles divide in a cell-cycle dependent manner and the cell-cycle progression is synchronized by a 12-h light/12-h dark cycle (Suzuki et al., 1994). *C. merolae* does not have a rigid cell wall and thus it is relatively easy to homogenize the cells and extract the cellular contents. In addition, the nuclear and organelle genomes have been completely sequenced (Ohta et al., 1998, 2003; Matsuzaki et al., 2004; Nozaki et al., 2007). The nuclear genome size is extremely small with little genetic redundancy (16.5 Mb; 4,775 protein-coding genes). These features have facilitated omics analyses on a variety of cellular processes in photosynthetic eukaryotes, such as organelle division and inheritance (Miyagishima et al., 1998, 1999, 2003; Nishida et al., 2003; Yagisawa et al., 2007, 2009, 2012, 2013; Fujiwara et al., 2009, 2010; Yoshida et al., 2009, 2010; Imoto et al., 2011, 2013), circadian gating of cell-cycle progression (Miyagishima et al., 2014) and metabolism (Imamura et al., 2008; Moriyama et al., 2014) in photosynthetic eukaryotes.

Nuclear transformation in *C. merolae* is performed as follows. The parental strain is a uracil-auxotrophic strain, which has a point mutation in the chromosomal *URA5.3* gene (*URA*) (strain M4; Minoda et al., 2004) or in which the *URA* gene is completely deleted (Taki et al., 2015). To produce stable transformants, a transgene conjugated with the *URA* selection marker is integrated into a chromosome of the M4 strain by homologous recombination (*URA*-M4 selection system; Imamura et al., 2010; Fujiwara et al., 2013a). In order to perform gene knockout, the *URA* selection marker is used to disrupt the target locus by integration via homologous recombination (**Supplementary Figure S1**).

Until now, however, no transformation selection marker other than *URA* has become available, which has largely limited the application of genetic modification (e.g., multiple gene knockout, knock-in and integration have been infeasible). Therefore, we have sought to develop a second transformant selection marker. *C. merolae* cells are sensitive to chloramphenicol (CP), an inhibitor of protein synthesis in bacteria, mitochondria and chloroplasts, which has been widely used for the selection of transformants in other organisms (Minoda et al., 2004). Zienkiewicz et al. (2015) reported that the introduction of chloramphenicol transferase (*CAT*), a CP-resistance gene, confers CP resistance on *C. merolae* cells. However, the *CAT* gene was randomly integrated into the nuclear genome in that study. In addition, the usage of the *CAT* section maker for the genetic modification of *C. merolae* was not evaluated.

In this study, we have developed a method of manipulating the *C. merolae* nuclear genome based on homologous recombination and isolation of single transformant clones using the *CAT*-CP selection system. In addition, we have succeeded in producing a double transformant by using a combination of the *URA*-M4 and *CAT*-CP systems. This combined system will greatly facilitate the genetic modification of *C. merolae*. In addition, while we were preparing this paper, a method of integrating the *CAT* gene into *C. merolae* chloroplast was reported (Zienkiewicz et al., 2016). Thus, the combination of these newly developed techniques should enhance our capacity to genetically modify *C. merolae*.

## Materials and Methods

### Algal Culture

The wild-type *C. merolae* 10D (NIES-3377), HA-cyclin 1 and HA-cyclin 1/FLAG-CDKA strains were maintained in 2x Allen’s medium (Allen, 1959) in 60 mL tissue culture flasks (TPP) with agitation at 120 rpm under continuous white light (30 μmol/m^2^s) at 42°C. The uracil auxotrophic mutant M4 was maintained in MA2 (Ohnuma et al., 2008) medium supplemented with uracil (0.5 mg/mL) and 5-fluoroorotic acid monohydrate (0.8 mg/mL).

### Preparation of DNA Fragments for Transformation

The primers used in this study are listed in Supplementary Table S1. Transformation of the *CAT* marker was performed with DNA fragments that were prepared as follows. The 1 to 180 nucleotides in the *C. merolae APCC orf* encoding the chloroplast-transit peptides (60 amino acids) and the downstream nucleotides of *C. merolae* β-tubulin *orf* (200 bp, βt3′) were amplified by PCR with the primer sets No. 1/No. 2 and No. 3/No. 4, respectively, using *C. merolae* genomic DNA as the template. The *CAT orf* was amplified by PCR with the primer set No. 5/No. 6, with pC194 as the template (pC194, Gene ID: 4594904; encoding *Staphylococcus aureus* chloramphenicol acetyltransferase). In order to construct the plasmid pD184-CAT, the sequence encoding the APCC chloroplast-transit peptide, the *CAT orf*, and the downstream sequence of β-tubulin were cloned into the pD184-O250-EGFP-URACm-Cm (Fujiwara et al., 2013a), which was amplified by PCR with the primer set No. 7/No. 8, using the InFusion Cloning Kit (TAKARA). The DNA fragments that were used as linear vectors to produce *CAT*, *CAT*-1500, *CAT*-500, *CAT*-350, *CAT*-200, *CAT*-100, and *CAT*-50 strains were amplified by PCR with pD184-CAT as the template and the primer sets No. 9/No 10, No. 11/No. 12, No. 13/No. 14, No. 15/No. 16, No. 17/No. 18, No. 19/No. 20, and No. 21/No. 22, respectively.

The HA-cyclin 1 strain was prepared as follows. To construct pCYC1, the *CYC1 orf* flanked with the 1,500-bp upstream and 2,000-bp downstream genomic sequences was amplified by PCR with the primer set No. 23/No. 24 using genomic DNA as the template, and then it was cloned into the pGEM-T Easy vector (Promega). The *URA* selection marker (Fujiwara et al., 2013a), which was amplified by PCR with the primer set No. 25/No. 26 and pD184-O250-EGFP-URA_Cm-Cm_ as the template, was cloned into a pCYC1 vector amplified by PCR with the primer set No. 27/No. 28. Then the 3x HA-coding sequence (TACCCATACGATGTTCCTGACTATGCGGGCTATCCCTATGACGTCCCGGACTATGCAGGATACCCTTATGACGTTCCAGATTACGCT), which was amplified by PCR with the primer set No. 29/No. 30 and pBSb-THA (Ohnuma et al., 2008) as the template, was cloned into the pCYC1-URA vector amplified by PCR with the primer set No. 31/No. 32. The linear DNA vector, which consists of 1,500 bp of *CYC1* upstream, *3xHA-CYC1 orf*, 200-bp of *CYC1* downstream, *URA* and 1,800-bp of *CYC1* downstream, was amplified by PCR with M13 forward/reverse primers (TAKARA) and pHA-CYC1-URA as the template, and it was used for transformation of the *C. merolae* M4 strain.

The HA-cyclin 1/FLAG-CDKA strain was prepared as follows. To construct pCDKA, the *CDKA orf* flanked with the 1.8-kbp upstream and the 0.7-kbp downstream genomic sequences was amplified by PCR using the primer set No. 33/No. 34 and genomic DNA as the template, then was cloned into the pGEM-T Easy vector. The *CAT* selection marker, which was amplified by PCR with the primer set No. 35/No. 36 and pD184-CAT as the template, was cloned into the pCDKA vector amplified by PCR with the primer set No. 37/No. 38. Then the 3xFLAG-coding sequence (ATGGACTACAAAGACCATGACGGTGATTATAAAGATCATGACATCGATTACAAGGATGACGATGACAAG) was cloned into the pCDKA vector using the InFusion Cloning Kit by PCR with the primer set No. 39/No. 40 and pCAT-CDKA as the template. The linear DNA vector, which consists of -1,800 to -799 (the number is from the *CDKA* start codon) of *CDKA*, the *CAT* selection marker, -798 to -1 of the *CDKA orf*, *3xFLAG-CDKA* and the ∼0.7-kb downstream sequence of *CDKA*, was amplified by PCR with the primer set No. 41/No. 42 using pCAT-FLAG-CDKA as the template, and was then used for transformation of the HA-cyclin 1 strain.

### Transformation of *C. merolae*

The transformation was performed according to Ohnuma et al. (2008) with modifications. The wild-type strain (10D) was used as the parental strain for the *CAT* strains. To prepare cells for transformation, the cells were diluted in 50 mL of MA2 medium supplemented with uracil (0.5 mg/mL, MA2U) to give a concentration of OD750 = 0.3, incubated under continuous light (100 μmol/m^2^s) with aeration (600 mL ambient air/min) for ∼19 h, and then harvested by centrifugation (2,000 *g* for 5 min) after addition of Tween-20 to the culture at a final concentration of 0.002%. The cell pellet was suspended in 270 μL of MA2 medium for transformation. DNA vectors are introduced into *C. merolae* cells by the polyethylene glycol (PEG)-mediated protocol. To prepare 60% (w/v) of PEG4000 solution, 0.6 g of PEG4000 (Aldrich, #81240) was dissolved in 450 μL of MA2 medium at 95°C for 10 min. After dissolution, the PEG4000 solution was kept at 42°C on a heat block until use. A 4-μg of each linear DNA vector was prepared in 90 μL of water. 90 μL of vector solution, 10 μL of 10x transformation (TF) solution (400 mM (NH_4_)_2_SO_4_, 40 mM MgSO_4_, 0.3% H_2_SO_4_) and 100 μL of the PEG4000 solution were mixed well by pipetting in a 1.5 mL tube. Then 25 μL of the cell suspension was added to 200 μL of the TF-DNA-PEG4000 mixture and vigorously inverted 3–4 times and then immediately transferred to 40 mL of MA2U medium for incubation under continuous light (100 μmol/m^2^s) with aeration (300 mL ambient air/min) for 1 day. Then the cells were concentrated into 2 mL of MA2U medium by centrifugation at 1,500 *g* for 5 min. The cells were grown in a 24-well plate (TPP Techno Plastic Products AG) under continuous white light at 42°C in ambient air supplemented with 5% CO_2_ for 2–3 days. CP was added to the culture to select CP-resistant transformants and the culture was incubated for ∼10 days. The CP-resistant transformants were washed with CP-free MA2U medium, serially diluted and then spotted on starch beds on a MA2U gellan gum plate. Starch beds and MA2U gellan gum plates were prepared as described in Imamura et al. (2010) with minor modifications (Fujiwara et al., 2013a). The plates were incubated in a 5%-CO_2_ incubator for 2 weeks until colonies appeared. The colonies were transferred to starch beds on a new MA2U plate according to Fujiwara et al. (2013a). The occurrence of the homologous recombination events in the chromosomal CMD184C region in the *CAT* strains was examined by PCR with the primers No. 43 and No. 44.

To produce the HA-cyclin 1 strain, the M4 strain was transformed with the linear DNA vector prepared as described above. The occurrence of the homologous recombination event in the *CYC1* locus was examined by PCR with the primers No. 45 and No. 46. To produce the HA-cyclin 1/FLAG-CDKA strain, the HA-cyclin 1 strain was transformed with the linear DNA vector prepared as described above. The CP-resistant HA-cyclin 1/FLAG-CDKA cells were selected in MA2U medium supplemented with 150 μg/mL of CP. The colony was isolated as described above. The homologous recombination event in the *CDKA* locus was examined by PCR with the primers No. 47 and No. 48.

### Immunoblotting

Five μg of cellular total protein was separated in each lane by SDS-PAGE with a 10% acrylamide gel and then transferred to a PVDF membrane. The membrane was blocked with 5% skim milk in TBS-T (10 mM Tris -HCl, pH 7.5, 150 mM NaCl, 0.1% Tween 20). The anti-HA antibody (clone 16B12, Biolegend) was used to detect HA-cyclin 1 at a concentration of 0.5 μg/mL. The anti-DYKDDDDK (FLAG) tag antibody (clone 1E6, Wako) was used to detect FLAG-CDKA at a concentration of 1 μg/mL. An HRP-conjugated anti-mouse IgG antibody (Thermo Scientific) was used as a secondary antibody at a dilution of 1:20,000. The signal was detected by ECL Prime Western Blotting Detection Reagent (GE Healthcare) and an Image Quant LAS 4000 mini (GE Healthcare).

### DNA Gel Blotting

Probes to detect CMD184C and *CAT* were prepared using a DIG-PCR labeling kit (Roche) with the primer sets No. 49/No. 50 and No. 51/No. 52, respectively. The extraction, digestion, electrophoresis and transfer to a nitrocellulose membrane of the genomic DNA were performed as described in Fujiwara et al. (2013b). The hybridization was performed in PerfectHyb hybridization solution (TOYOBO) with each probe at a concentration of 50 ng/mL. The DNA band was detected with a DIG Nucleic Acid Detection Kit (Roche) and Image Quant LAS 4000 mini.

### Growth Curves

To measure growth rate of the D-sfGFP, HA-cyclin 1 and HA-cyclin 1/FLAG-CDKA strains, these strains were diluted to an OD750 of 0.4 in MA2 medium and grown for a week beforehand to prepare log-phase cells in the same culturing condition. Then cells were inoculated in a 15 mL of MA2 medium at an OD750 of 0.2 in a 30 mL Erlenmeyer flask (IWAKI/PYREX), with agitation at 130 rpm under continuous white light (80 μmol/m^2^s) at 42°C. The change in OD750 was monitored every 2 days. The D-sfGFP strain was previously prepared by the integration of a superfolder *gfp* gene conjugated with the *URA* selection marker into a chromosome of the M4 strain (Fujiwara et al., 2015).

## Results

### Nuclear Transformation of the *CAT* Selection Marker by Homologous Recombination and the Isolation of Single Transformant Clones

**FIGURE 1:**
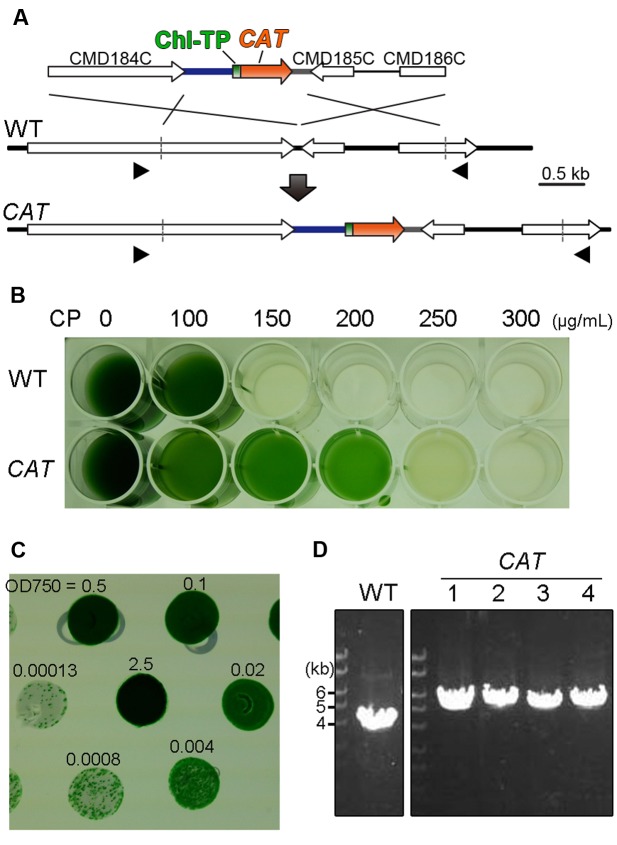
**Development of the selection system using chloramphenicol and the *CAT* selection marker.** **(A)** Schematic diagram of *CAT* gene insertion into the intergenic region between CMD184C and CMD185C by homologous recombination. The first line indicates the introduced liner DNA vector and the second line indicates the genomic structure of the parental wild-type (WT) strain. For the efficient expression of *CAT* (orange) and the translocation of the CAT protein into the chloroplast, the 600-bp upstream flanking sequence of the *APCC orf*, the sequence encoding the chloroplast-transit peptide (Chl TP) of APCC and the 200-bp downstream flanking sequence of the β-tubulin *orf* were utilized as the promoter (blue), the chloroplast-transit peptide (green) and the polyadenylation signal sequence (gray), respectively. The third line indicates the expected genomic structure of the *CAT* strain. The arrowheads indicate the positions of the PCR primers No. 43 and No. 44 used in **(D)**. The exact positions and sequences are indicated in Supplementary Table S1. **(B)** The selection of CP-resistant transformants in the liquid medium supplemented with a series of concentrations of CP. **(C)** Single-colony isolation of transformed cells on the solidified (gellan gum) medium without CP. Transformed cells, which were selected in the liquid medium supplemented with CP, were washed with CP-free medium. Then cells were serially diluted as indicated and spotted on the plate. **(D)** PCR analysis of the independent *CAT* transformants was performed to confirm the homologous recombination event. The WT strain was used as a negative control. The positions of PCR primers No. 43 and No. 44 are shown in **(A)** and the exact positions and sequences are indicated in Supplementary Table S1. The predicted size of the PCR product is 5.4 kb for *CAT*-targeted insertion and 3.8 kb for *CAT*-off-targeted insertion, respectively.

In order to validate the application of the *CAT* gene as a selection marker for the nuclear transformation of *C. merolae* by homologous recombination, we constructed a *CAT* selection marker cassette consisting of the *APCC* promoter, a chloroplast-transit peptide, the *CAT orf* and the β-tubulin 3′ *utr*. The *APCC* promoter was chosen for two reasons: the *APCC* (CMO250C) gene was highly expressed under light condition (Fujiwara et al., 2015) and it was shown that the ∼600-bp of upstream flanking sequence is functional as a promoter (Watanabe et al., 2011). The nucleotide sequence encoding the chloroplast-transit peptide (60 N-terminal amino acids of APCC; Watanabe et al., 2011) was fused to the *CAT orf* of *Staphylococcus aureus* (Gene ID: 4594904) in order to translocate the CAT protein into the chloroplast stroma, because the target of chloramphenicol is the chloroplast ribosome (Zienkiewicz et al., 2015). In order to integrate the *CAT* selection marker cassette into the convergent intergenic region of CMD184C and CMD185C as a neutral chromosomal locus by homologous recombination, the cassette was flanked with ∼1.5-kb chromosomal sequences (**Figure 1A**). The intergenic region of CMD184C and CMD185C is very short and therefore does not likely possess any promoter activities that would potentially affect gene expression in its vicinity. The growth curves of *C. merolae* stains, in which the *URA* selection marker was inserted into the region, were indistinguishable from that of the parental strain (Fujiwara et al., 2013a). In addition, the mRNA and protein levels of sfGFP expressed from the region are almost the same as those expressed from another chromosomal locus (the upstream region of *URA5.3* locus). Thus, the intergenic region of CMD184C and CMD185C is regarded as a neutral chromosomal locus (Fujiwara et al., 2015).

After transformation, the *CAT* transformants were selected by incubation in liquid MA2 medium supplemented with CP instead of an MA2 gellan gum plate supplemented with CP because we failed in our effort to grow transformants on the plate supplemented with CP. In order to determine the appropriate concentration of CP in the liquid selection, the wild-type and the transformants were cultured in the liquid medium supplemented with a series of concentrations of CP (0, 50, 100, 150, 200, and 250 μg/mL) for 10 days. As a result, the wild-type died while the transfomant (*CAT*) grew in MA2 supplemented with 150 or 200 μg/mL of CP (**Figure 1B**).

In order to obtain single transformant clones, cells in the liquid medium supplemented with 200 μg/mL of CP were serially diluted and then spotted onto a CP-free MA2 plate. After incubation for 2 weeks, transformed colonies appeared (**Figure 1C**). We confirmed that the *CAT* marker was integrated into the intergenic region of CMD184C and CMD185C in the four clones tested by colony PCR analysis (**Figure 1D**).

### 200-bp Homologous Arms Are Required and 500-bp Arms Are Sufficient for a Targeted Single-Copy Insertion of the *CAT* Selection Marker into the Nuclear Genome

**FIGURE 2:**
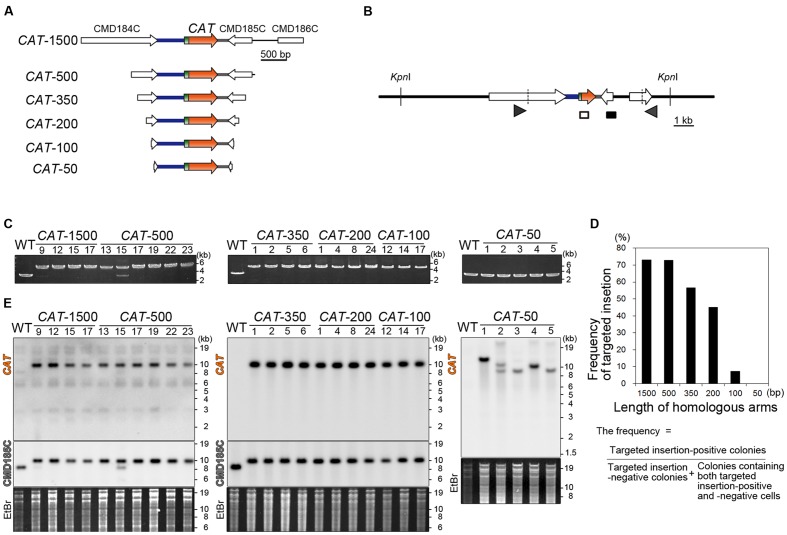
**Length of the homologous arms sufficient for the nuclear insertion of the *CAT* selection marker by homologous recombination.** **(A)** A schematic diagram of the linear DNA vector of the *CAT* selection marker flanked with the ∼1,500-, ∼500-, 350-, 200-, 100-, or 50-bp genomic sequences. The genomic sequences were flanked so as to target the *CAT* marker into the intergenic region between CMD184C and CMD185C. The respective linear DNA vectors were named *CAT*-1500, *CAT*-500, *CAT*-350, *CAT*-200, *CAT*-100, and *CAT*-50. **(B)** A schematic diagram of the predicted *CAT* insertion into the intergenic region between CMD184C and CMD185C by homologous recombination. The arrowheads indicate the positions of the PCR primers used in **(C)**. The diagram includes the positions of the probes (open and closed boxes) and the restriction enzyme *Kpn*I sites that were used in the DNA gel blot analysis **(D)**. The hybridization probes shown as open and closed boxes recognize the *CAT orf* and CMD185C *orf*, respectively. **(C)** PCR analysis of the independent *CAT* transformant was performed to confirm the homologous recombination events. The wild-type (WT) strain was used as a negative control. The positions of the PCR primers No. 43 and No. 44 are shown in **(B)** and the exact positions and sequences are indicated in Supplementary Table S1. The predicted size of the PCR product is 4.6 kb for the positive strains and 2.8 kb for the negative strains. Note that the *CAT*-500-#15 transformant was a mixture of *CAT*-targeted cells and *CAT*-off-targeted cells. **(D)**. A bar graph showing the effect of length of homologous arms on targeting efficiency. Targeting frequency is the percentage of targeted insertion-positive colonies. *n* = 37, 33, 30, 41 and 56 colonies of *CAT*-1500, 500, 350, 200, 100, and 50 transformants, respectively. **(E)** DNA gel blotting of the *CAT*-1500, *CAT*-500, *CAT*-350, *CAT*-200, *CAT*-100, and *CAT*-50 strains. The WT strain was used as a negative control. The genomic DNA extracted from the respective strain was digested with *Kpn*I. The DNA fragment containing the introduced DNA was detected by two probes that recognize the *CAT orf* and CMD185C *orf*, respectively. The predicted size of the DNA fragment, which is detected with the *CAT orf* probe, is 10.3 kb for the *CAT*-inserted chromosome. The predicted size of the DNA fragment, which is detected with the CMD185C *orf* probe, is 10.3 kb for the *CAT-*inserted chromosome and 8.1 kb for the WT chromosome, respectively. The upper left and upper right figures are the results obtained with the *CAT orf* probe. The middle left figure is the result with the CMD185C *orf* probe. A portion of the ethidium bromide (EtBr)-stained gel is shown as the loading control.

Thus far, 1∼1.5-kb homologous arms have been flanked with the *URA* transformation marker to target a transgene or knockout a gene by homologous recombination. In this study, in order to evaluate the effect of length of homologous arms upon targeting efficiency, we introduced the *CAT* selection marker flanked with 1500, 500, 350, 200, 100, or 50-bp homologous arms (**Figure 2A**). The transformants that resulted were respectively named *CAT*-1500, 500, 350, 200, 100, and 50. After transformation and isolation of single transformant colonies, the occurrence of homologous recombination was examined by PCR (**Figures 2B,C**; only a part of the results are shown, in **Supplementary Figure S2**, a part of negative results in the *CAT*-1500, 500, 350, 200, and 100 are shown). In 27 out of 37 colonies (73%) of the *CAT*-1500, in 24 out of 33 colonies (73%) of *CAT*-500, in 17 out of 30 colonies (57%) of *CAT*-350, in 18 out of 40 colonies (45%) of the *CAT*-200, and in 3 out of 41 colonies (7%) of the *CAT*-100 transformants, integration of the *CAT* marker by homologous recombination was detected, whereas no targeting by homologous recombination was detected in all (56 colonies) of the *CAT*-50 transformants (**Figure 2D**). Two bands were detected by PCR and DNA gel blot analysis in the *CAT*-500 #15 transformant (**Figures 2C,E**). The upper band and the lower band were derived from *CAT*-targeted cells and *CAT*-off-targeted cells (*CAT* was randomly integrated into a chromosome without homologous recombination event), respectively. Such colonies are probably derived from a mixture of *CAT*-targeted cells and *CAT*-off-targeted cells (these colonies were counted as negative transformants in the above). Therefore, *CAT*-targeted cells can be isolated from the mixture by additional single colony isolation.

The DNA gel blot analysis demonstrated single-copy-integration of the *CAT* marker without any off-target insertion in the *CAT*-1500, *CAT*-500, *CAT*-350, *CAT*-200 and *CAT*-100, whereas undesired and multiple integration of the *CAT* marker occurred in *CAT*-50 (**Figure 2D**). Thus, 200-bp homologous arms are required for targeted insertion of the *CAT* marker in the *CAT*-CP selection system in *C. merolae.* In addition, ∼500-bp homologous arms are desired to increase the frequency of the targeted insertion.

### Application of the *CAT*-CP Selection System to Produce a Double Gene-Knock-In *C. merolae* Clone by a Combination of the *URA*-M4 and *CAT*-CP Selection Systems

As an example of the application of *CAT*–CP selection system, we designed a double knock-in *C. merolae* strain in which HA-tagged cyclin 1 (*C. merolae* gene ID: *CYC1*/CML219C) and FLAG-tagged cyclin-dependent kinase A (*CDKA*/CME119C) are expressed from their respective chromosomal loci. *C. merolae* cyclin 1 is a functional homolog of the cyclin D/E (G1 cyclin) found in other eukaryotes (Miyagishima et al., 2014). Plant CDKA, which is related to mammalian Cdk1, is constitutively expressed during the cell cycle in *Arabidopsis thaliana* (Scofield et al., 2014). A crucial role of plant CDKA is believed to be control of the transition from the G1 to S phase, which is a cell-size checkpoint, by association with cyclin D (Sablowski and Dornelas, 2014). In addition, CDKA also associates with cyclin A (an S-phase cyclin) and then cyclin B (an M-phase cyclin) sequentially, and regulates the progression of both the S and M phases (Scofield et al., 2014). CDKA activity regulates progression of the cell cycle, while the contribution of CDKA to M-phase progression, which is regulated in parallel by CDKB (an M-phase specific plant CDK), still remains unclear. Thus, monitoring of the activity of cyclin 1-CDKA complex, the binding of cyclin 1 and CDKA and the posttranslational modifications in cyclin 1 and/or CDKA in *C. merolae* will advance our understanding of the relationship between cellular growth and cell-cycle progression in photosynthetic eukaryotes.

**FIGURE 3:**
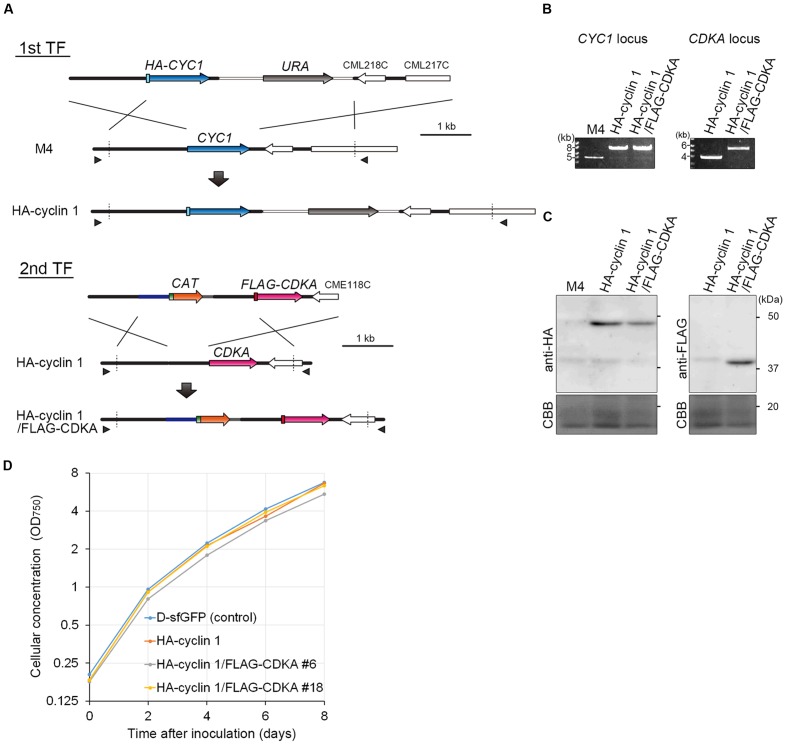
**Production of the HA-cyclin 1 and FLAG-CDKA expressing double-knock-in strain.** **(A)** Schematic diagram of the knock-in of *HA-CYC1* and *FLAG-CDKA* into the chromosomal *CYC1* and *CDKA* loci by homologous recombination. The first line indicates the linear DNA vector that was transformed into the M4 strain. The second and third lines indicate the genomic structure of the parental M4 strain and resultant HA-cyclin 1 strain, respectively. The fourth line indicates the linear DNA vector that was transformed into the HA-cyclin 1 strain. The fifth and sixth lines indicate the *CDKA* locus of the parental HA-cyclin 1 strain and the resultant HA-cyclin 1/FLAG-CDKA strain, respectively. The arrowheads indicate the positions of the PCR primers used in **(B)**. **(B)** PCR analysis of the HA-cyclin 1 and the HA-cyclin 1/FLAG-CDKA strains, confirming the occurrence of homologous recombination events. The M4 strain was used as a negative control. The positions of the primer sets No. 45/No. 46 and No. 47/No. 48 are shown in **(A)** and the exact positions and sequences are indicated in Supplementary Table S1. The predicted size of the PCR product amplified from the *CYC1* locus is 6.8 kb for the HA-cyclin 1 strain and 4.0 kb for the M4 strain. The predicted size of the PCR product amplified from the *CDKA* locus is 4.5 kb for the HA-cyclin 1/FLAG-CDKA strain and 2.9 kb for the HA-cyclin 1 strain. **(C)** Immunoblotting with the anti-HA and the anti-FLAG antibodies. The predicted sizes of HA-cyclin 1 and FLAG-CDKA proteins are 48 and 40 kDa, respectively. **(D)** Growth curves of the D-sfGFP strain (an sfGFP expresser was used as a control), the HA-cyclin 1 and the HA-cyclin 1/FLAG-CDKA strains.

In this study, we produced an HA-cyclin 1 and FLAG-CDKA double knock-in strain by a two-step transformation. First, *HA-CYC1* was knocked-in by the *URA*-M4 selection system. To this end, a DNA fragment consisting of the *CYC1* upstream genomic sequence, *HA-CYC1 orf*, *URA* marker and *CYC1* downstream genomic sequence was introduced into the *C. merolae* M4 strain (**Figure 3A**, 1st TF). The resulting strain was named the HA-cyclin 1 strain. Then, we knocked-in *FLAG-CDKA* into the HA-cyclin 1 strain. A DNA fragment consisting of the *CDKA* upstream genomic sequence, *FLAG-CDKA orf*, *CAT* selection marker and *CDKA* downstream genomic sequence was introduced into the HA-cyclin 1 strain (**Figure 3A**, 2nd TF). The resulting strain was named the HA-cyclin 1/FLAG-CDKA strain.

The PCR analysis showed that the both *HA-CYC1* and *FLAG-CDKA* were integrated into the chromosomal *CYC1* and *CDKA* loci, respectively, in the HA-cyclin 1/FLAG-CDKA strain (**Figure 3B**). In addition, the immunoblotting with the anti-HA antibody and the anti-FLAG antibody demonstrated that both the HA-cyclin 1 and FLAG-CDKA proteins were expressed in the HA-cyclin 1/FLAG-CDKA strain (**Figure 3C**). Thus, the combination of the *URA*-M4 and *CAT*-CP selection systems enables production of double-knock-in strains by a two-step transformation in *C. merolae*. The *C. merolae* genome encodes single copies of cyclin 1 and CDKA and the transformants were shown to proliferate normally (**Figure 3D**), suggesting that both the HA-cyclin 1 and FLAG-CDKA are fully functional in *C. merolae*.

## Discussion

Efficient gene targeting and stable transgene expression are major advantages for the transformation system in *C. merolae* compared to other eukaryotic algae. However, no transformation selection marker other than *URA* has been generally available. Accordingly, genetic modification efforts have been limited. Although the *CAT* gene is reported to function as a transformation marker in *C. merolae*, integration of the marker into a desired nuclear locus by homologous recombination has not been achieved (Zienkiewicz et al., 2015). In addition, the usage of the *CAT*-CP selection system in gene targeting in *C. merolae* has not been reported. In this study, the utility of the *CAT-*CP selection system is demonstrated for example, for the production of a double knock-in strain (**Figure 3**).

Although the *URA*-M4 system requires the M4 strain or another *URA* mutant, the *CAT-*CP selection system does not require any specific strains and is applicable, for example, to the wild-type strain. Differences of the biological features between the uracil-auxotrophic mutants and the WT strain have been poorly characterized. Previous yeast studies have reported that gene expression patterns in auxotrophic mutants are different from those of a complementation strain even in a nutrient-rich medium (Brem et al., 2002). It is also likely inadequate to directly compare uracil auxotrophic mutants and their transformants in *C. merolae*. Therefore, it is preferable to use the wild-type strain and wild-type-derived transformants that are produced by the *CAT*-CP selection system. Furthermore, the combination of the *CAT*-CP and *URA-*M4 selection systems enables multiple modifications of chromosomal loci, such as double knock-in (**Figure 3**), double disruption and the integration of many transgenes into chromosomal neutral loci. These are quite important in the genetic modification in *C. merolae* because sexual reproduction has not been reported in this organism. When a simultaneous double transformation method is developed in future, it will be a great time-saver. Its efficiency likely depends on the nature of the targeting genes or loci. In addition, we showed that 200-bp homologous arms are required and 500-bp arms are sufficient for targeted insertion in the *CAT*-CP selection system (**Figure 2**). Thus, either upstream or downstream homologous arm can be shortened to 200 bp. For example, this is useful for excluding repetitive DNA elements from targeting DNA vectors because repetitive elements prevent accurate detection of targeted insertion on PCR or DNA gel blot analyses (Aoyama et al., 2005). In addition, shortening the homologous arms will facilitate the preparation of linear DNA vectors for introduction and PCR identification of the positive transformant colonies.

During the preparation of this paper, a transformation method for the *C. merolae* chloroplast genome was reported (Zienkiewicz et al., 2016). The authors succeeded in targeted the *CAT* gene insertion into the chloroplast genome by homologous recombination, although the targeting of a transgene into the chloroplast genome or modification of a chloroplast-encoded gene was not reportedly tested. Further combination of the nuclear *URA*-M4 and *CAT*-CP systems with the chloroplast *CAT*-CP system should facilitate additional studies in *C. merolae*, for example on the interactions between the nuclear and chloroplast genomes.

## Author Contributions

Conceived and designed the experiments: TF, MO, RO, SH, and S-YM. Performed the experiments: TF. Analyzed the data: TF. Contributed reagents/materials/analysis tools: TF, MO, and TK. Wrote the paper: TF, S-YM.

## Conflict of Interest Statement

The authors declare that the research was conducted in the absence of any commercial or financial relationships that could be construed as a potential conflict of interest.

## Supplementary Material

FIGURE S1 **A procedure for production of transgene-inserted and gene knockout strains by using uracil-auxotrophic-mutants as parental strains. (A)** Schematic diagrams of the uracil-auxotrophic mutants and respective liner DNA vectors for transgene insertion and gene knockout. Two lines of uracil-auxotrophic mutants, M4 and T1, of *C. merolae* have been used to produce transformant strains. One is the M4 strain in which one adenine base is inserted in the *URA*/CMK046C gene coding region (gray), resulting in a translational frameshift in the 3′ half of the *orf* (Minoda et al., 2004). The other is the T1 strain in which the *URA* locus including the ∼900-bp flanking upstream region and the *orf* (dashed line) is deleted (Taki et al., 2015). Right lower schemas show linear DNA vectors to be introduced to the M4 or T1 strain and to produce (1) transgene (magenta)-inserted strains and (2) gene-knockout strains. As shown in (1), the upstream and downstream sequences of a neutral chromosomal locus (black bars) were flanked with a set of a transgene and the *URA* selection marker so as to target the neutral chromosomal locus by homologous recombination. As shown in (2), the upstream and downstream sequences of a target gene (stripe bars) were flanked with the *URA* selection marker so as to knockout the gene. Nu, nucleus. **(B)** Schematic diagrams of insertion of a set of a transgene (magenta) insertion and the *URA* selection marker (gray) into a neutral chromosomal locus by homologous recombination. The first line indicates the introduced liner DNA vector and the second line indicates the genomic structure of the parental M1 or T1 strain. The third line indicates the expected genomic structure of a transgene-inserted strain. The black bars indicate the flanking upstream and downstream sequences of a neutral chromosomal locus. Nu, nucleus; Mt, mitochondrion; Ch, chloroplast. **(C)** Schematic diagrams of a gene knockout by replacement with the *URA* selection marker (gray). The first line indicates the introduced liner DNA vector and the second line indicates the genomic structure of the parental M1 or T1 strain. The third line indicates the expected genomic structure of a gene knockout strain. The stripe bars indicate the flanking upstream and downstream sequences of a target gene (green).

FIGURE S2 **DNA agarose gel electrophoresis showing colony-direct PCR analysis of the independent *CAT*-1500, *CAT*-500, *CAT*-350, *CAT*-200, and *CAT*-100 transformants.** A part of negative results as well as positive results were shown. The WT strain was used as a negative control. The predicted size of the PCR product is 4.6 kb for the *CAT*-targeted transformants and 2.8 kb for the *CAT*-off-targeted transformants and the WT strain. The results of the *CAT*-1500-#24, *CAT*-500-#1, *CAT*-350-#34, *CAT*-200-#31 and *CAT*-100-#12 transformants were positive, indicating that *CAT*-targeted insertion has occurred. The results of the *CAT*-1500-#22,*CAT*-500-#3, *CAT*-350-#35, *CAT*-200-#30, *CAT*-100-#11 and *CAT*-100-#13 transformants were negative, indicating that *CAT*-off-targeted (random) insertion had occurred. Note that PCR analyses of the *CAT*-1500-#23 and *CAT*-500-#2 transformants failed. The positions of the PCR primers No. 43 and No. 44 are shown in **Figure 2B** and the exact positions and sequences are indicated in Supplementary Table S1.
