# Supplementary figures and images for: Development of a Double Nuclear Gene-Targeting Method by Two-Step Transformation Based on a Newly Established Chloramphenicol-Selection System in the Red Alga Cyanidioschyzon merolae
Source: Front Plant Sci. 2017 Mar 14;8:343. doi: 10.3389/fpls.2017.00343 (PMC5348525; doi:10.3389/fpls.2017.00343)

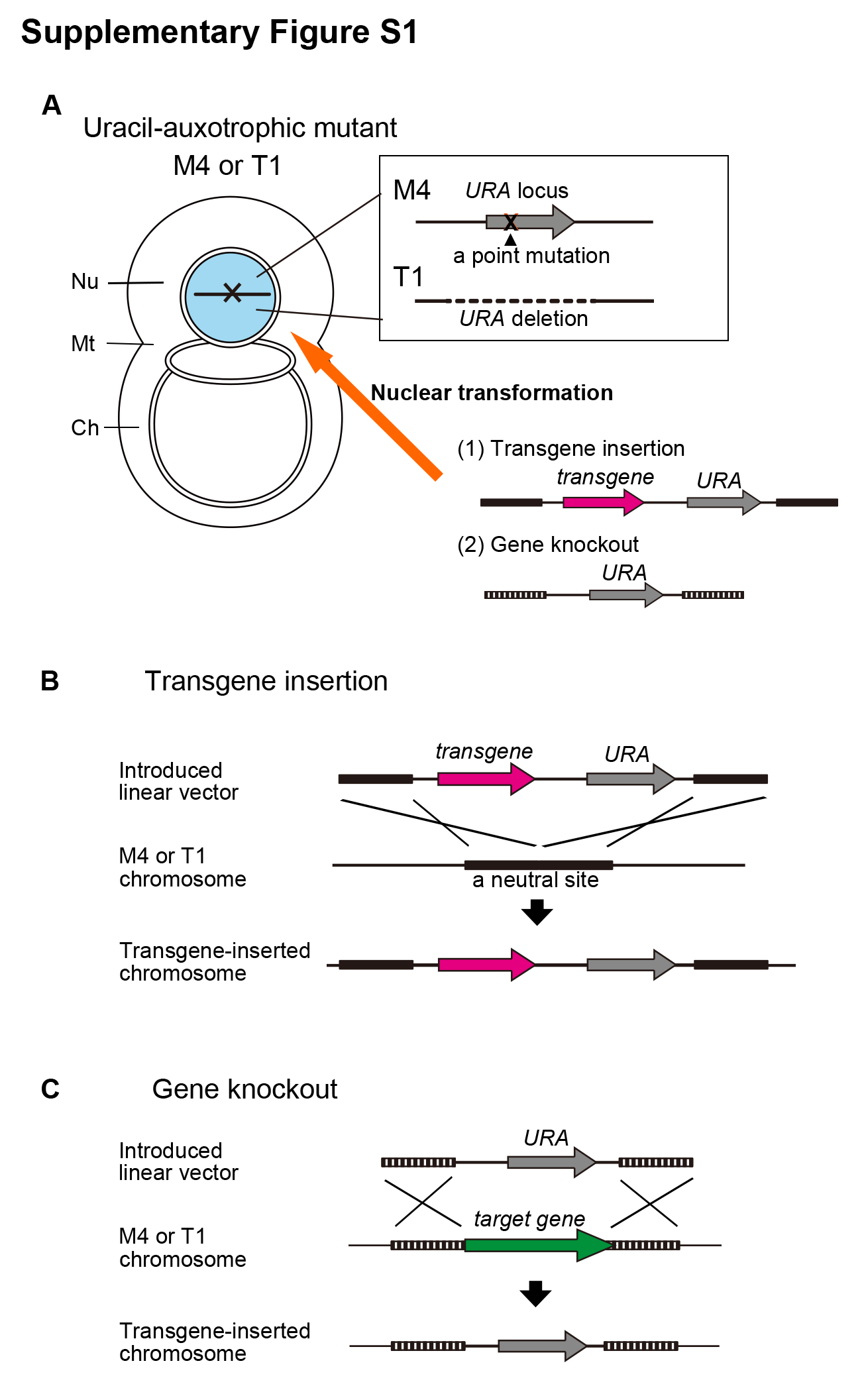

Supplement: FIGURE S1 — A procedure for production of transgene-inserted and gene knockout strains by using uracil-auxotrophic-mutants as parental strains. (A) Schematic diagrams of the uracil-auxotrophic mutants and respective liner DNA vectors for transgene insertion and gene knockout. Two lines of uracil-auxotrophic mutants, M4 and T1, of C. merolae have been used to produce transformant strains. One is the M4 strain in which one adenine base is inserted in the URA/CMK046C gene coding region (gray), resulting in a translational frameshift in the 3′ half of the orf (Minoda et al., 2004). The other is the T1 strain in which the URA locus including the ∼900-bp flanking upstream region and the orf (dashed line) is deleted (Taki et al., 2015). Right lower schemas show linear DNA vectors to be introduced to the M4 or T1 strain and to produce (1) transgene (magenta)-inserted strains and (2) gene-knockout strains. As shown in (1), the upstream and downstream sequences of a neutral chromosomal locus (black bars) were flanked with a set of a transgene and the URA selection marker so as to target the neutral chromosomal locus by homologous recombination. As shown in (2), the upstream and downstream sequences of a target gene (stripe bars) were flanked with the URA selection marker so as to knockout the gene. Nu, nucleus. (B) Schematic diagrams of insertion of a set of a transgene (magenta) insertion and the URA selection marker (gray) into a neutral chromosomal locus by homologous recombination. The first line indicates the introduced liner DNA vector and the second line indicates the genomic structure of the parental M1 or T1 strain. The third line indicates the expected genomic structure of a transgene-inserted strain. The black bars indicate the flanking upstream and downstream sequences of a neutral chromosomal locus. Nu, nucleus; Mt, mitochondrion; Ch, chloroplast. (C) Schematic diagrams of a gene knockout by replacement with the URA selection marker (gray). The first line indicates the intro [file Image_1.tif]

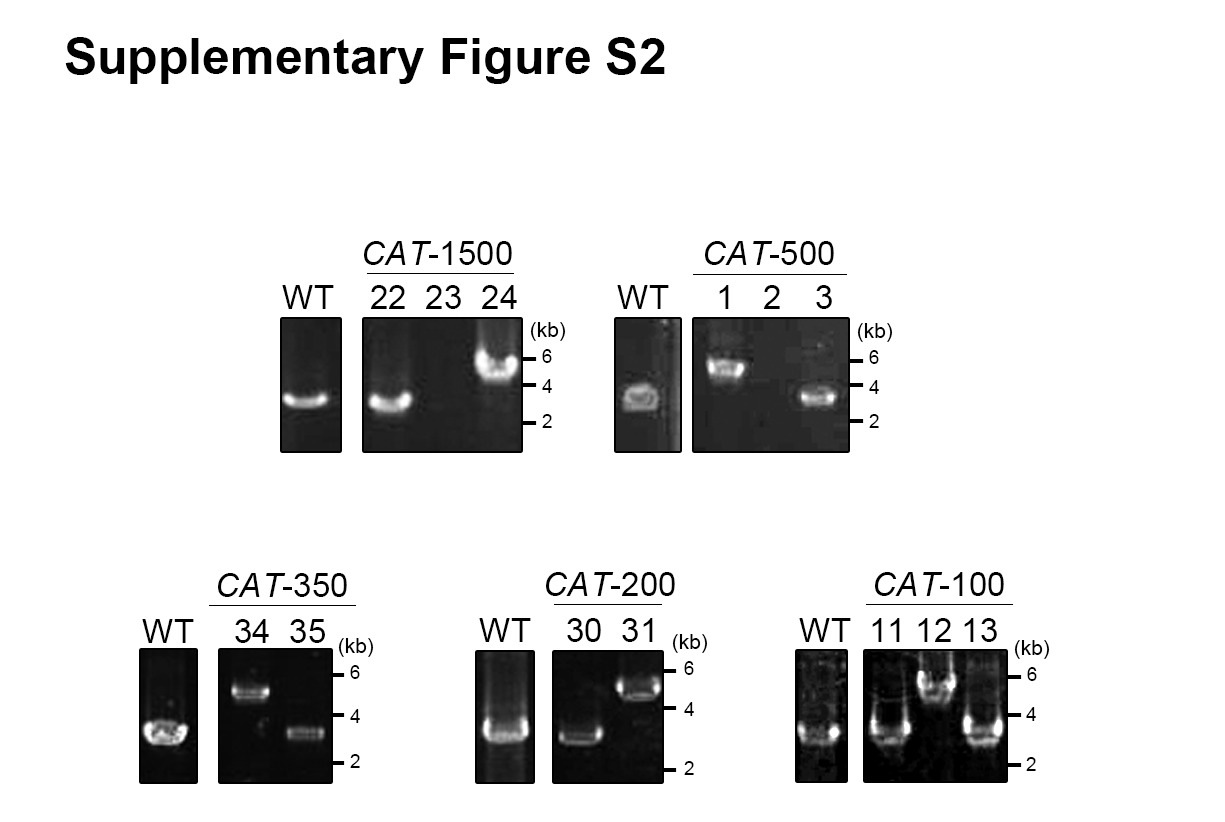

Supplement: FIGURE S2 — DNA agarose gel electrophoresis showing colony-direct PCR analysis of the independent CAT-1500, CAT-500, CAT-350, CAT-200, and CAT-100 transformants. A part of negative results as well as positive results were shown. The WT strain was used as a negative control. The predicted size of the PCR product is 4.6 kb for the CAT-targeted transformants and 2.8 kb for the CAT-off-targeted transformants and the WT strain. The results of the CAT-1500-#24, CAT-500-#1, CAT-350-#34, CAT-200-#31 and CAT-100-#12 transformants were positive, indicating that CAT-targeted insertion has occurred. The results of the CAT-1500-#22,CAT-500-#3, CAT-350-#35, CAT-200-#30, CAT-100-#11 and CAT-100-#13 transformants were negative, indicating that CAT-off-targeted (random) insertion had occurred. Note that PCR analyses of the CAT-1500-#23 and CAT-500-#2 transformants failed. The positions of the PCR primers No. 43 and No. 44 are shown in Figure 2B and the exact positions and sequences are indicated in Supplementary Table S1. [file Image_2.tif]
