# Supplementary material for: Development of a Double Nuclear Gene-Targeting Method by Two-Step Transformation Based on a Newly Established Chloramphenicol-Selection System in the Red Alga Cyanidioschyzon merolae
Source: Front Plant Sci. 2017 Mar 14;8:343. doi: 10.3389/fpls.2017.00343 (PMC5348525; doi:10.3389/fpls.2017.00343)
Supplement: Supplementary file 3 [file Table_1.pdf]

Supplementary Table 1. Primers used in the current study

1	APCC(1)Fapc	cttcgttcgttgaccATGTTCGTTCAGACCAGTTTCTTT
2	APCC(180)R	ATCATTCGCAACGCCAGATG
3	B-TUB(+1)	TAAACTAGCTATTTATCTGGTACATATCATTCAT
4	B-TUB(+200)Rd184	tcgctgagcggtttcACACTTTTTGCCTGCACAAGTTTTCGTAC
5	CAT(1)Fapcc	ggcgttgcgaatgatATGAACTTTAATAAAATTGATTTAGACAATTGG
6	CAT(650)taaRbt	taaatagctagtttaTAAAAGCCAGTCATTAGGCCTATC
7	APCC(-1)R	GGTCAACGAACGAAGAAACACAGAGAACAAAGATAT
8	D184(+28)F	GAAACCGCTCAGCGACCAAGCGAC
9	D184(1270)F	ACACGAATCACACGGTGCTG
10	D184(+1448)R	TTGCCGATAACGCAGAAGAGA
11	D184(1200)F	CGCCTTCTCCTGGACGAGTAC
12	D184(+1400)R	CCAGAGCCCTACCGGCA
13	D184(2229)F	CGGGCCGGAGAACATCTTTATTTTCGG
14	D184(+525)R	GCCCTGACACGCGCTCGAG
15	D184(2409)F	CACTTTTCAGGAAATTGTACGAGCCCT
16	D184(+375)R	TGCTTTTTTCGCGAACGACCCC
17	D184(2559)F	GGCTCGCATGGGTAAGTTCAG
18	D184(+225)R	CGCTGTCGTGCAGCGC
19	D184(2659)F	TACAAGGAACCAGTTGAGGGTGTC
20	D184(+125)R	TTGCGTCGCGTGAACTACTTGG
21	D184(2709)F	GCCGAAGACGACGCTCCGAG
22	D184(+75)R	CACGCGACGAGGTGGCGAG
23	CYC1(-1500)F	GCTCGAATACACTCACTTGAGAG
24	CYC1(+2000)r	CTATCGAACGCCTGTTCCTGAA
25	URA(-897)F	GAACTGAGGGGCGAACGCA
26	URA(+471)R	CCCTAGCAGCTGACTGTATC
27	CYC1(+200)Rura	ttcgcccctcagttcTTCGATGAGGTCTGGTGGC
28	CYC1(+201)Fura	agtcagctgctagggACTCGAACGCATGTGCTCG
29	HA-F	TACCCATACGATGTTCCTGACTATG
30	HA-R	AGCGTAATCTGGAACGTCATAA
31	CYC1(15)Rha	aacatcgtatgggtaCGTTTCAACCAGCATGACCA
32	CYC1(+4)Fha	gttccagattacgctCTGGTTGAAACGAAGGGATCTG
33	CDKA(-1800)F	ACCCTGAACGGTAAGATGTTGC
34	CDKA(+1759)R	TCAGTTGTGTACGGCGCTTT
35	APCC(-600)F	CGACGAGAACGTATAAGGAGTGCGCACG
36	B-TUB(+200)R	ACACTTTTTGCCTGCACAAGTTTTCGTACG
37	CDKA(-799)Rapcc-600	tatacgttctcgtcgTATGCCTGTATCGAGCCCGG
38	CDKA(-798)Fbt+200	gcaggcaaaaagtgtGCGACACGCATCAAACCACA
39	CDKA(-1)R_FLAG	gtctttgtagtccatGGTTCCCTGAAAATCCGCAG
40	FLAG-CDKA(4)F	ATGGACTACAAAGACCATGACGGTGATTATAAAGATCATGACAT
41	CDKA(-1760)F	TGAGGCTCTGGGGTTGCAGAAC
42	CDKA(+675)R	GTACGATGCTCGGTTGCGCCT
43	D184(1111)F	GAGGTTCCTGAAGAAGGACCG
44	D184(+1500)R	GACGCATCCGCTCGTGTC
45	CYC1(-1800)F	CCTCTTGCAACTTTTCCCTGC
46	CYC1(+2199)R	ATACACTCGAACAGGCCCTC
47	CDKA(-1820)F	CGAAGGGTCATGCGTGGAG
48	CDKA(+1039)R	CGGTGTCCGCTTCTGTGC
49	D184(+68)F	GTCGCGTGGTCTCGTCG
50	D184(+367)R	TCGCGAACGACCCCGAAG
51	CAT(1)F	ATGAACTTTAATAAAATTGATTTAGACAATTGG
52	CAT(250)R	CTAACTTATCCCAATAACCTAACTCTCC

Lowercase letters indicate adaptor sequences for In-Fusion reaction.
